# CDX2 loss in colorectal cancer cells is associated with invasive properties and tumor budding

**DOI:** 10.1038/s41598-025-07278-x

**Published:** 2025-07-06

**Authors:** Nils Bodmer, Kristin Uth, Rina Mehmeti, Cansaran Saygili Demir, Deborah Stroka, Nassim Ghaffari-Tabrizi-Wizsy, Alessandro Lugli, Olivier de Wever, Inti Zlobec, Mario P. Tschan

**Affiliations:** 1 Institute of Tissue Medicine and Pathology, University of Bern, Murtenstrasse 31, 3008 Bern, Switzerland; 2 Graduate School for Cellular and Biomedical Sciences, University of Bern, Uni Mittelstrasse, Mittelstrasse 43, 3012 Bern, Switzerland; 3 Department of Visceral Surgery and Medicine, Inselspital, Bern University Hospital, University of Bern, Bern, Switzerland; 4 Department for BioMedical Research, University of Bern, Bern, Switzerland; 5 Division of Immunology, Research Unit CAM Lab, Otto Loewi Research Center, Medical University of Graz, Graz, Austria; 6 Cancer Research Institute Ghent (CRIG), Ghent University, Ghent, Belgium; 7 Laboratory for Experimental Cancer Research, Ghent University, Ghent, Belgium; 8 Institute of Tissue Medicine and Pathology, University of Bern, Murtenstrasse 31, Room L310, CH-3008 Bern, Switzerland; 9 Institute of Tissue Medicine and Pathology, University of Bern, Murtenstrasse 31, Room L523, CH-3008 Bern, Switzerland; 10 Lunaphore Technologies , Tolochenaz, Switzerland

**Keywords:** Colorectal cancer, CDX2, Tumor budding, Cell migration, EMT, Colorectal cancer, Cell migration

## Abstract

In colorectal cancer (CRC), tumor buds (TB) are observed histologically as single tumor cell or small tumor cell clusters located mainly at the advancing tumor edge. TB are a marker of poor prognosis and correlate with metastatic disease in CRC patients. They often lack expression of CDX2 and overexpress markers involved in epithelial-mesenchymal transition (EMT). We evaluated the function of CDX2 in CRC proliferation and migration using CRISPR/Cas9 technology and demonstrated a possible link to tumor dissociation and tumor budding. Knocking out CDX2 in CRC cell lines significantly increased migration. Importantly, the observed phenotypes could be rescued by re-expressing CDX2 and by specific CRISPR synergistic activation mediator (SAM) of endogenous CDX2 in CDX2 low expressing CRC cell lines. Multiplex immunofluorescence (mIF) analysis of primary tumor regions compared to TB in a CDX2-positive CRC patient sample as well as patient derived xenografts (PDX) revealed significantly lower CDX2 expression and correlating E-cadherin levels in TB compared to primary tumor regions, in both models. Accordingly, increased invasiveness of CRC *CDX2* knockout cells was seen in *ex ovo* xenografts. Taken together, our results provide further insight into the function of CDX2 in preventing CRC cell migration, tumor budding and tumor aggressiveness.

## Introduction

Colorectal cancer (CRC) is reported to be the third most common, and the second deadliest cancer worldwide according to statistics by the World Health Organization^1^. The main metastatic site remains the liver due to the rich blood supply system that connects both organs. The homeobox protein CDX2 has gained considerable attention over the last years as a potential prognostic biomarker of poor outcome in patients with CRC^2^. Although typically expressed in gastrointestinal tumors, up to 20% of colorectal cancers show a reduction or complete absence of CDX2 protein expression, as detected by immunohistochemistry^3^. These findings are of clinical relevance, as it has been suggested that patients with stage II CRC (i.e. lymph node metastasis negative) with CDX2 loss represent a particular high-risk group that may benefit from adjuvant therapies^4^.

The mechanism by which CDX2 is lost has not been fully elucidated. Interestingly, the mutation rate of CDX2 is reported to be exceedingly low^5^. On the other hand, DNA promoter methylation and histone acetylation have been described as important epigenetic mechanisms of transcriptional repression and loss of CDX2 protein^6,7^. CDX2 loss is often seen in cases of chromosomal instability and is expressed in a more heterogeneous manner throughout the tumor tissue. In fact, closer examination of CDX2 staining shows that cells at the invasion front (advancing edge) of the cancer often lose expression^2^.

Tumor buds are defined as single tumor cells or small tumor cell clusters of up to four cells detached from the main tumor body on histological sections^8^. Over the last decade, the process of “tumor budding” has gained tremendous attention due to its consistent and reproducible unfavorable prognostic effect^9,10^. In fact, in 2016, the International Tumor Budding Consensus Conference (ITBCC) took place to reach a consensus on tumor bud scoring and reporting as well as to establish the clinical scenarios under which budding plays a relevant role in CRC management^8^. Tumor budding occurs in a multitude of different cancer types, e.g. lung, head and neck, pancreas, and breast cancer, thus has far reaching clinical importance^11–14^.

Tumor buds are associated with loss of the cell-to-cell adhesion molecule E-cadherin, leading to translocation of β-catenin to the nucleus, and upregulation of numerous proteins involved in extracellular matrix degradation as well as invasion and migration of tumor cells^15,16^. In fact, co-expression of epithelial and mesenchymal markers in a subset of tumor buds speaks for a partial EMT state in these cells^17^.

We hypothesize that downregulation of CDX2 promotes colorectal cancer cell migration, invasion and tumor budding predisposing patients to poor prognosis and therapy response due to increased metastasis. We aim to elucidate the future potential of CDX2 as a therapeutic target to counteract CRC cell dissemination in patients.

## Results and discussion

### Loss of CDX2 increases migration and invasion of colorectal cancer cells

**Fig. 1:**
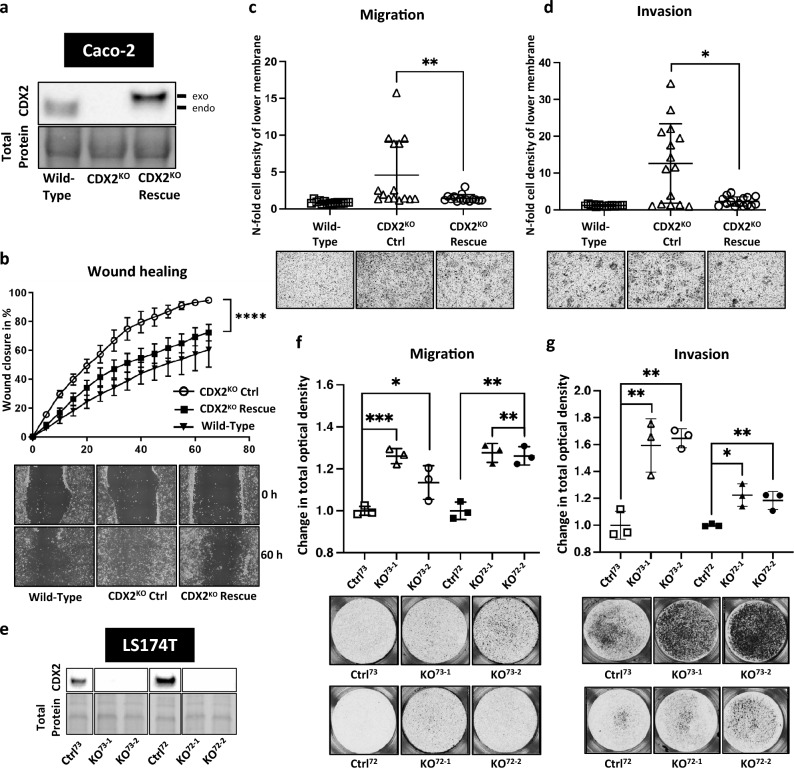
Deletion of CDX2 in colorectal cancer (CRC) cells promotes cancer cell migration and its exogenous expression in Caco-2 CDX2 knockout cells is able to rescue this phenotype. (**a**) Western blot analysis confirming CDX2 knockout and rescued Flag-CDX2 in Caco-2 cells (endo = endogenous, exo = exogenous). (**b**) Wound healing assay of CDX2 rescued compared to CDX2 knockout cells as control (mean + SEM). (**c**) Transwell migration assay of cells in (**a**). (**d**) Transwell invasion assay of cells in (a). Statistical evaluation using two-way ANOVA and nonparametric Mann–Whitney U were applied for wound healing and transwell migration/invasion assays, respectively, on PRISM software (GraphPad Software, San Diego). (**e**) Western blot confirming CDX2 deletion in four LS174T CDX2 knockout clones (KO^73–1^, KO^73–2^, KO^72–1^, KO^72–2^) compared to two controls (Ctrl^73^, Ctrl^72^) (relevant sections of the whole blot). (**f**) Transwell migration assay of cells in (**e**). (**g**) Transwell invasion assay of cells in (**e**). Statistical evaluation using two-way ANOVA and unpaired T-test was done for wound healing and transwell migration/invasion assays, respectively, on PRISM software (GraphPad Software, San Diego). n ≥ 3. *p ≤ 0.05; **p ≤ 0.01; ***p ≤ 0.001; ****p ≤ 0.0001 in all graphs.

To investigate whether CDX2 loss can impact CRC proliferation and migration *in vitro*, we generated LS174T and CaCo2 CDX2 knockout cells^18,19^. We transduced these cell lines with two lentiviral CRISPR/Cas9 constructs encoding two independent gRNAs targeting exon 1 of the *CDX2* gene. Expression of both gRNAs resulted in *CDX2* knockout phenotypes in all colon cancer cell clones (Fig. 1a, e; Supp. Fig. S1a, b).

To test how CDX2 affects cellular proliferation, we performed cell counting and colony formation assays with control and *CDX2* knockout CRC cells. Our results revealed significantly lower cell numbers of CaCo2 and LS174T *CDX2* knockout cells in comparison to the parental cells after 4 days in culture (Suppl. Fig. S1c, f) in alignment with reduced colony formation (Suppl. Fig. S1d, e, g, h). Since knocking out *CDX2* did not cause increased cell proliferation, we analyzed the migratory potential of these *CDX2* knockout cell lines using wound healing and transwell migration and invasion assays. We found significantly increased migratory ability of CaCo2 *CDX2* knockout cell clones in comparison to parental tracking the migration of the cells by live cell imaging (Fig. 1b). Since the growth behavior of LS174T cells did not allow to generate a well-defined scratch in a cell monolayer, we performed transwell migration assays with these cells and analyzed the migration rate through the porous membrane of the insert. Similar to CaCo2 *CDX2* knockout cells, LS174T *CDX2* knockout clones showed significantly increased migratory activity compared to their respective control clones (Fig. 1f). In addition, knocking out CDX2 significantly increased cellular invasion in matrigel (Fig. 1g).

To investigate whether the observed increased motility phenotype is specifically due to CDX2 modulation we performed an add-back experiment in CaCo2 CDX2 knockout cells. We overexpressed CDX2 in CaCo2 *CDX2* knockout cells using a lentiviral construct encoding for FLAG-tagged human *CDX2* (Fig. 1a; Suppl. Fig S1a). Firstly, re-expressing CDX2 restored the proliferation rate as found in the CaCo2 wildtype cells (Suppl. Fig. S1i). Secondly, the migratory potential assessed in wound healing and transwell migration was reversed to near-wildtype levels in CDX2-rescued CaCo2 cells (Fig. 1b, c). Thirdly, re-expressing CDX2 also reversed the increased invasion rate caused by CDX2 depletion (Fig. 1d).

At first glance, the above data of decreased proliferation versus increased cell motility in CDX2 CRC knockout cells seem in contrast to previous findings demonstrating that knocking down CDX2 leads to increased proliferation in CaCo2 and HT29 colon cancer cells^19^. This discrepancy might be based on using CDX2 null instead of knockdown cells where residual CDX2 might be responsible for a contradictory phenotype. This discrepancy could also be caused by hypoxic conditions^19^. Strikingly, our results showed a significant increase in the migration and invasion ability of *CDX2* knockout in two different CRC cell lines. These results indicate that loss of CDX2 expression indeed promotes a more aggressive cancer phenotype, consistent with the existing literature on the well-documented correlation of CDX2 loss with unfavorable patient outcome^4,21^. Our findings are in agreement with previously published data showing that CDX2 has a negative effect on colon cancer motility and dissemination^22^. Consequently, CDX2 likely is an important modulator of colorectal cancer progression.

### Specific (re-)activation of endogenous CDX2 in CDX2 negative colorectal cancer cells resulted in decreased cell motility

**Fig. 2:**
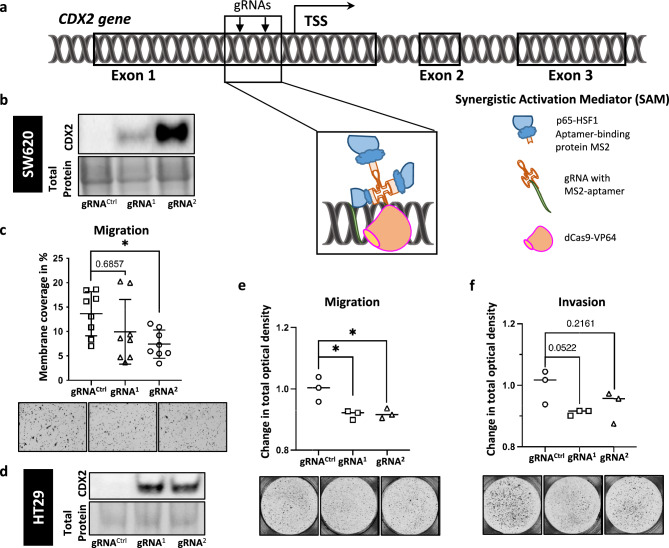
Endogenous CDX2 activation in SW620 and HT29 cell lines causes significant decrease of cell motility. (**a**) Schematic representation of the CRIPSR/dCas9 SAM system in SW620 and HT29 colon cancer cell lines. Two gRNAs bind the MS2 aptamer attached to p65-HSF1 (enhancer of transcriptional activation) and guide dead (**d**) Cas9 to *CDX2* promoter regions upstream of the transcriptional start site (TSS). (**b**) Western blot confirmation of CDX2 protein expression in SW620 cells. (**c**) Transwell migration assay of SW620 cells in (**b**), n > 3. (**d**) Western blot confirmation of CDX2 protein expression in HT29 cells. (**e**) Transwell migration assay of HT29 cells in (**d**). (**f**) Transwell invasion of HT29 cells in (**d**). n = 3 in (**e**) and (**f**). Nonparametric Mann–Whitney U was applied for transwell migration assay with n > 3, whereas unpaired T-test was applied for transwell migration and invasion assay with n = 3, on PRISM software (GraphPad Software, San Diego). *p ≤ 0.05.

To further validate our findings from *CDX2* knockout and overexpression experiments in a more physiologically relevant model, we activated endogenous *CDX2* in CDX2 negative SW620 and HT29 colon cancer cell lines. To this end we designed several gRNAs targeting the proximal 5’UTR region of *CDX2* and transduced them together with the lentiviral CRISPR/dCas9 Synergistic Activation Mediator (SAM) and the helper activator complex, MS2-p65-HSF1 into SW620 and HT29 cells (Fig. 2a). Two of the gRNAs tested allowed for activation of endogenous CDX2 expression in SW620 and HT29 cells as shown by western blotting and immunohistochemistry (Fig. 2b, d; Suppl. Fig. S2a). Activation of endogenous CDX2 in SW620 cells resulted in an increase of cellular proliferation and colony formation (Suppl. Fig. S2b, c, d) supporting our previous CDX2 knockout data in CRC cells showing decreased proliferation and colony formation upon gene knockout. Importantly, activation of endogenous CDX2 expression mediated by the CRISPR/SAM system significantly attenuated transwell migration in both CRC cell lines. The effect of transwell migration attenuation was CDX2 dose dependent, which varied between the two different gRNAs and the two cell lines (Fig. 2c, e). Further, the assessment of transwell invasion in HT29-SAM cells showed a similar pattern upon *CDX2* activation and thus supports our findings from above analysis (Fig. 2f).

As hypothesized, activation of endogenous CDX2 expression using a CRISPR/dCas9 synergistic activation mediator (SAM) system allowed of specific activation of *CDX2*^23^ paralleled by a decrease in cell migration as well as invasion^19,21^. Of note, CDX2 reduces cellular motility in a dose-dependent manner that is in line with previous findings from our group that showed dose-dependent epigenetic silencing of *CDX2* and thus supporting a quantitative relevance^7^. Together, reactivation of CDX2 expression in CDX2-negative colorectal cancer reduces tumor cell migration and invasion^19^.

### Low CDX2 and E-Cadherin expression levels are associated with tumor budding at the primary site and at the liver metastatic site of CRC patients

**Fig. 3:**
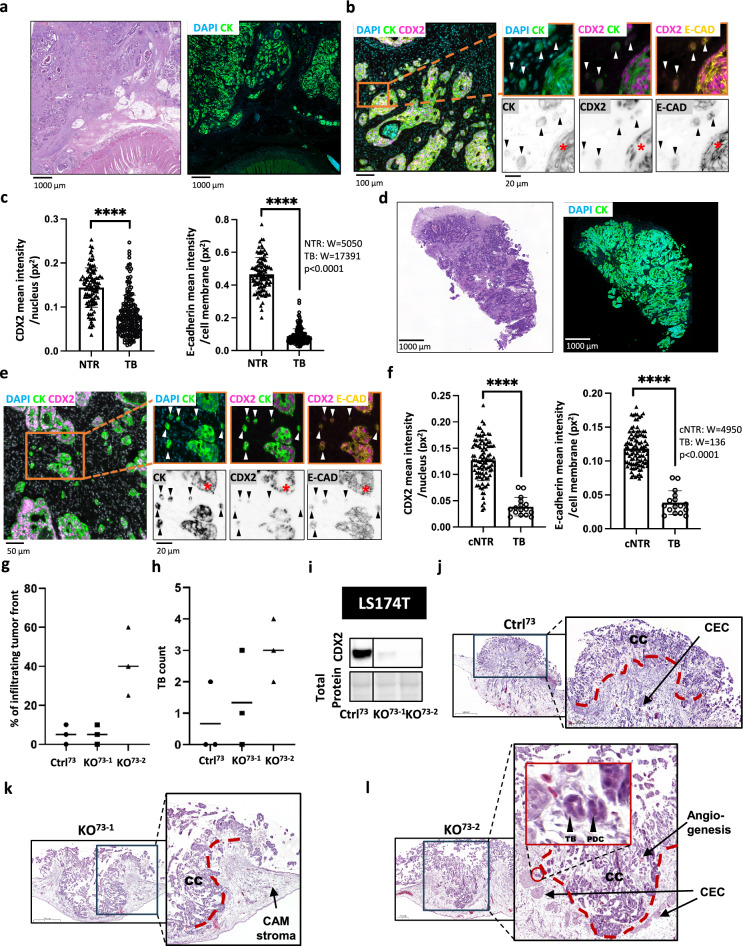
CDX2 and E-cadherin expression in tumor buds versus primary tumor in a CRC patient sample. (**a**, **d**) H&E staining of the whole slide image of a CRC patient sample and patient-derived xenograft (PDX) model, respectively. (**b**, **e**). The panels illustrate downregulation of cytokeratin (CK), CDX2 and E-cadherin (E-CAD) in tumor buds (indicated by arrowheads) compared to the nearest primary tumor regions (indicated by asterix). (**c**, **f**) Graphs show CDX2 and E-cadherin expression quantified in tumor buds versus epithelial cells in the nearest tumor region. (**c**) NTR, nearest tumor region n = 100, TB, tumor buds n = 186, ****p < 0.0001. (**f**) cNTR, cells in the nearest tumor region n = 99, TB, tumor buds n = 16, ****p < 0.0001. Wilcoxon signed-rank test was applied for statistical evaluation on PRISM software (GraphPad Software, San Diego). (**g**–**l**) Chorioallantoic membrane (CAM) assay of LS174T *CDX2* knockout clones vs. *CDX2* positive control. (**g**) Infiltrating margin analysis and (**h**) tumor bud count of cells incubated on CAM, stained for H&E. (**i**) Western blot confirmation of CDX2 knockout of cells used for CAM assay. (**j**–**l**) CAM tissue slides stained for H&E of one control (Ctrl^73^) and two *CDX2* knockout clones (KO^73–1^ and KO^73–2^); CEC: CAM epithelial cluster; CC: Cancer cells; TB: Tumor bud; PDC: poorly differentiated cluster.

By applying multiplex immunofluorescence on CRC patient and patient-derived xenograft (PDX) samples, we were able to explore an association between low CDX2 and E-cadherin levels, the latter being a well-established EMT marker, in tumor buds and corresponding nearest tumor region (Fig. 3a-f). Quantitative analysis showed that both CDX2 and E-cadherin levels were significantly lower in tumor buds compared to their adjacent tumor regions in a CRC patient sample (Fig. 3c). Interestingly, in the PDX model, representing metastasis of CRC in the liver, we were able to identify tumor buds as well. Similarly to the primary CRC sample, we observed significantly lower CDX2 and E-cadherin levels in tumor buds compared to the cells in the nearest tumor region (Fig. 3f). In line with these findings *in vivo*, we also observed significantly reduced mRNA and protein levels of E-Cadherin in LS174T *CDX2* knockout compared to wild-type cells *in vitro* (Suppl. Fig. S3a, b, c).

These findings suggest that downregulation of CDX2 is a predisposition for detached and disseminated CRC cells from the main tumor, such as tumor buds. Tumor budding has been described as an independent prognostic factor and associated with lymph node metastasis in CRC^8^. Previous studies have also linked decreased CDX2 expression to a more aggressive phenotype and poorer prognosis in CRC^22,23^. Additionally, the decrease in E-cadherin levels suggests an ongoing epithelial-mesenchymal transition (EMT) process within tumor buds, which may contribute to their invasive properties. Moreover, CDX2 has also been described as an inhibitor of EMT^24^. However, these findings are limited by the sample size and the reliance on immunocompromised mice for the PDX model, which may not fully recapitulate human tumor microenvironment. Nevertheless, this proof-of-concept analysis demonstrated the feasibility of using multiplex immunofluorescence in analyzing expression of multiple markers in tumor buds, laying the groundwork for broader application in larger cohorts. Future studies should also explore the mechanisms by which CDX2 modulates EMT and tumor budding. Such studies may provide valuable insights into the development of selective therapies targeting tumor bud formation and inhibiting CRC metastasis.

### Increased invasive behavior of CDX2 knockout cells *ex ovo*

To further test our hypothesis that loss of CDX2 contributes to a more aggressive behavior of colorectal cancer cell lines, we made use of the chorioallantoic membrane assay (CAM)^18,19^. The chorioallantois of the chicken egg provides a physiological environment for the colorectal cancer cells to grow and thus represents a relevant tool to study the behavior of colorectal cancer cells^25^. The application of LS174T CDX2 knock-out clones (Fig. 3g-l) onto the chicken egg revealed substantial differences in aggressiveness to its relative control clone (Ctrl73). LS174T cells showed a strongly increased tissue infiltration in one CDX2 knockout clone compared to control cells (Fig. 3g). Additionally, we were able to detect tumor budding (TB) in the tissue slides created from CAM assay cell grafts (Fig. 3l). Tumor budding count suggested an increased frequency for clone KO73-2 compared to control (Fig. 3h). In summary, depleting CDX2 in CRC cells increased their aggressive phenotype in a xenograft model.

3D models show that loss of CDX2 expression is a key feature of enhanced invasive tumor cells^9,24^. In patient samples as well as in the 3D models, invasive cells show a significant reduction of CDX2 expression and epithelial cell markers such as E-cadherin^13^. These results suggest a transformation of cells with epithelial characteristics to mesenchymal-like cells, which then are able to penetrate tissue as first step towards the formation of metastases. Our findings are supported by the observation that CDX2 expression and cell polarization is lost in the tumor buds but restored in the resulting metastasis^26^. Further, we showed that the CAM assay provides an opportunity to model tumor budding for colorectal cancer cells^25^. The CAM assay is, compared to *in vivo* animal models, a more simplistic, rapid, resource-saving model that can be used as a valuable preliminary step prior to more complex animal models^25^. Further studies are needed to elucidate the role of CDX2 in preventing an EMT-like phenotype which is closely related to tumor budding and associated with an increased risk for colorectal cancer patients^7,20,24^.

We provided further evidence of a key role of CDX2 in preventing tumor cell dissemination and possibly tumor budding using state-of-the-art CRISPR/Cas9 and /dCas9 technology to manipulate CDX2 expression in colon cancer cells. We also showed that loss of CDX2 is associated with tumor budding and leads to a more aggressive phenotype in several 3D models. Additionally, the newly generated cell models will facilitate more physiological relevant studies in the future, aiming at deciphering the function of CDX2 in colon cancer pathology. In conclusion, our data hint to a substantial role of CDX2 in tumor progression in 2D and 3D models, which should be further investigated.

## Materials and methods

### Cell culture

LS174T (ATCC) cells were maintained in Dulbecco′s Modified Eagle′s Medium (DMEM, D6046, Sigma) supplemented with 10% Fetal Bovine Serum (FBS, Sigma) and 2 mM L-Glutamine (G7513, Sigma). CaCo2 (ATCC) cells were grown in Minimum Essential Medium Eagle (EMEM, M4526, Sigma) supplemented with 20% FBS and 2 mM L-Glutamine and SW480 and SW620 (ATCC) cells were kept in RPMI-1640 (RPMI, R8758, Sigma) supplemented with 10% FBS. HT29 were kept in Mc Coy’s 5A (M8403, Sigma) supplemented with 10% FBS and 2 mM L-Glutamine. All cells were cultured in a humidified atmosphere of 5% CO_2_.

### Lentiviral vectors

*CRISPR-Cas9 CDX2 knockout* two gRNA sequences (HGLibA_08899: CTACGGCGGTTACCACGTGG and HGLibA_08898: GACTGGAATGGCTACGCGCC) targeting the CDX2 gene were taken from the Human GeCKOv2 CRISPR knockout pooled library and cloned into the lentiCRISPR v2 vector (kindly provided by Feng Zhang, Addgene plasmid #52,961) using In-Fusion® Cloning (Takara Bio USA, Inc.) according to their instructions.

*CDX2 expression vector* Human CDX2 cDNA was amplified from the CMV-CDX2-WT plasmid kindly provided by Bert Vogelstein (Addgene plasmid #16,553). We added a Flag-sequence to the forward primer for tagging CDX2 and cloned the Flag-CDX2 fragment into pLV-EF1a-IRES_Neo backbone kindly provided by Tobias Meyer (Addgene plasmid #85,139) using In-Fusion® Cloning (Takara Bio USA, Inc.) according to their instructions.

*Synergistic Activation Mediator (SAM) system vectors* The following SAM system lentiviral plasmids were a gift from Feng Zhang: lenti-sgRNA-(MS2)_zeo backbone (Addgene plasmid #61,427), lenti-dCAS9-VP64_Blast (Addgene plasmid #61,425), and lentiMPH_v2 (Addgene plasmid #89,308). Four different gRNAs were designed using the Cas-Activator-Tool (http://sam.genome-engineering.org/database) and cloned into the lenti-sgRNA-(MS2)_zeo backbone. Two of the four CDX2 gRNA sequences (AGGGGAGGGAGGCTCGTGAG and GAGGAAGCTCTTAACAATAA) resulted in marked activation of endogenous CDX2 expression. We used a control gRNA cloned in the same (MS2) zeo backbone as negative control.

### Lentiviral vector preparation and cell transduction

293T cells for virus production were maintained in DMEM (Sigma-Aldrich, St. Louis, MO, USA), supplemented with 5% FBS, 1% penicillin/streptomycin (Sigma-Aldrich), and 1% Hepes (Sigma-Aldrich), and kept in 7.5% CO2 at 37 °C.

The following lentiviral vectors CRISPR-Cas9-CDX2-gRNA1 (HGLibA_08899), CRISPR-Cas9-CDX2-gRNA2 (HGLibA_08898) (Zhang lab), and pCas-Scramble CRISPR Vector (Cat#: GE100003, Origene) contained a puromycin antibiotic-resistance gene for selection of transduced mammalian cells. The Flag-CDX2 expression vector contained a hygromycin antibiotic-resistance gene for selection of transduced mammalian cells.

Lentiviral vectors for the SAM system, namely lenti-sgRNA-(MS2)_zeo, lenti-dCAS-VP64_Blast and lentiMPH_v2 contain a zeocin, blasticidin and hygromycin antibiotic-resistance gene, respectively, for selection of transduced mammalian cells.

Third generation lentiviral particles were produced by transient co-transfection of the packaging plasmids pMD.G (VSV-G), pMDLg/p.RRE (gag and pol), and pRSV-Rev (Rev gene), as well as the respective lentiviral transfer vector. Viral supernatant was harvested 48 h later. Cell lines were seeded in 6-well plates one day before the transduction was performed overnight using 500 μL viral supernatant and 8ug/ml polybrene per well. Four days after transduction the cells were selected with the respective antibiotics (Puromycin, 1.5 μg/ml; Hygromycin, 100–600 µg/ml; Blasticidin, 5 µg/ml; Zeocin, 200–1000 µg/ml). Where necessary single cell cloning was performed by serial dilution of cells in 96-well plate format.

### Western blot analysis

Cell lysates were prepared using 8 mM Urea in ELGA® water containing TBS-T and 0.5% (v/v) Triton X-100 supplemented with Protease Inhibitor (04,693,116,001; Roche Diagnostics, Rotkreuz, Switzerland). Protein concentrations were determined using a Bradford Assay (Bio-Rad Protein Assay; Bio-Rad, Cressier, Switzerland). 30-50 μg of total protein was mixed with loading buffer (4 × Laemmli Sample Buffer, Cat: #161–0747, BioRad) to a total volume of 15ul, loaded on Mini-Protean TGX Stain Free Gels (12%, 15-well, Cat. #456–8095; BioRad) and transferred to a PVDF membrane (0.2 μm PVDF, Cat. #170–4156; BioRad) using a Trans-Blot Turbo Transfer Pack (Mini format; BioRad). Membranes were blocked with 5% milk in 1X Tris buffered Saline with 0.1% Tween® (TBS-T)for at least 30 min. Blots were incubated with anti-CDX2 (1:5000 in 5% BSA in TBS-T; EPR2764Y, Sigma-Aldrich) and anti-E-Cadherin (1:1000 in 5% milk in TBS-T;14,472, Cell Signaling) over night at 4 °C followed by incubation with anti-rabbit or anti-mouse, respectively (1:10′000 in BSA; Cell Signalling Technology, Leiden, The Netherlands) for at least one hour at RT. Detection has been performed using ECL substrate (Clarity Western ECL Substrate, Cat. #170–5060; BioRad) and a ChemiDoc system (#731BR00765; BioRad). To quantify western results, protein expression was normalized to total protein expression using ImageJ software version 1.54d (www.imagej.net/ij/).

### Cell growth and colony formation assays

For trypan blue counting 4 × 10^4^ cells were seeded in duplicates in a 24-well plate and were counted every day using Neubauer chambers (Neubauer). For colony formation assays SW620 and LS174T cells were seeded in 6-well plates at a density of 1 × 10^3^ cells per well. CaCo2 cells were seeded at a density of 0.5 × 10^3^ cells per well. After 6 (SW620, CaCo2) or 10 days (LS174T) cell colonies have been fixed with 4% sterile-filtered PFA for 15 min and stained with 0.05% Crystal Violet (V5265, Sigma) in 30% EtOH for one hour. Plates have been washed two times with PBS, once with distilled water, then dried overnight. Colonies were counted using an Oxford Optronix Gelcount TM apparatus (Oxford Optronix, UK).

### Wound-healing assays

Wound healing: CaCo2 cells and the respective sublines were seeded in duplicates in 6-well plates (Sarstedt) at a density of 9.5 × 10^5^ /well. After 24 h a scratch was applied across the well using a pipette tip. Live cell imaging was performed on a Cell IQ2 cell imaging and analysis system (110,004; Chip-Man Technologies). Wound closure was determined using CellActivision software (Yokogawa Electric Corporation, www.yokogawa.com).

### Transwell migration/invasion assays

Transwell migration assay: The respective cells were seeded in 0%FBS Media one day prior to experiment at 2 × 10^6^ cells in a 10 cm dish. SW620, LS174T, HT29-SAM and CaCo2 cells were seeded at densities of 4 × 10^5^, 2 × 10^5^, 2 × 10^5^ and 5 × 10^4^ cells per transwell insert (TC for 24well, PET 8 µm, Sarstedt) in medium without FBS, respectively. Transwell inserts were then placed in a 24-well plate (Sarstedt, Suspension Plates), each well containing medium supplemented with 20% FBS as a chemoattractant for 48 h.

Transwell invasion assay: To assess the invasion capabilities of the following cell lines the transwell inserts were coated with 50 μλ Matrigel (Corning, Cat:5,236,014). CaCo2, LS174T and HT29-SAM cells were subsequently seeded at a density of 1 × 10^5^, 2 × 10^5^ and 2 × 10^5^ cells per insert, respectively (TC for 24well, PET 8 µm, Sarstedt). The inserts were placed in an empty 24-well plate and 550 µl medium were added to the well below. The media was supplemented with 40% FBS (as a chemoattractant) for a 40 h incubation of Caco2 cells and 20% FBS and 48 h for LS174T and HT29 SAM cells.

### The chick chorioallantoic membrane (CAM) assay

To perform the *ex ovo* chick chorioallantoic membrane assay (CAM assay) fertile white Lohmann chicken eggs (Schropper, Gloggnitz, Austria) were washed and incubated at 37.6 °C and 40–60% humidity. On day three of embryonic development (EDD3) eggs were carefully cracked and content transferred into sterile weighing boats (100 ml, 85 × 85x24 mm, VWR®, Avantor, Vienna, Austria) and covered with a sterile quadratic 10 × 15 mm petri dish (Bartelt, Graz, Austria) and further incubated under the same conditions as mentioned above. On EDD10, sterile silicone rings of 5 mm diameter were placed on the CAM of each embryo, avoiding placement directly on bigger blood vessels. The cells were then added at 1 × 10^6^ cells in 15ul Medium plus 5 µl Matrigel® (Corning® Matrigel® Basement Membrane Matrix, Phenol Red-Free, 10 mL, Product #356,237) in the center of each silicone ring. After another 72 h, on EED 13, the formed CAM tumors were photographed, excised and fixed in 4% PFA and paraffin embedded according to standard protocol. Further analysis was done by Haematoxylin/Eosin (H&E) staining and subsequent Immunohistochemistry (IHC). CAM fulfills the 3R principle (Replacement, Refinement and Reduction) by representing an alternative to animal models. The tumor scoring, budding analysis and margin characterization was performed by a trained pathologist based on the H&E slides.

### Primary patient sample

One CRC patient sample block was retrieved from the archives of the Institute of Tissue Medicine and Pathology, University of Bern. The corresponding diagnostic H&E slides were evaluated for budding and deemed appropriate for downstream analysis with multiplex immunofluorescence. Informed consent was obtained from the patient prior to surgery.

### Patient-derived Xenograft (PDX) models

Human CRC tumor tissues were obtained from consented patients at the University Hospital of Bern undergoing surgical colorectal resection. Informed consent was obtained prior to surgery in compliance with the local ethics regulations and under approval of local ethics commission. All animal experiments were carried out in accordance with relevant guidelines. Tumor samples were taken from within the tumor margin as well as from outside the tumor margin to be used as a non-malignant control. Tumor tissue samples were collected in basic medium. Tumor fragments were transplanted subcutaneously in immunocompromised mice (Rag2/Il2rg double knockout, C57BL/6NTac.Cg-Rag2^tm1Fwa^Il2rg^tm1Wjl^, obtained from Taconic Biosciences).

To implant the tumors, mice were anesthetized with 2% isoflurane at a flow rate of 2L/min and simultaneously administered the analgesic buprenorphine (Temgesic™) at a dose of 0.1 mg/kg. The PDX tumor was removed from mice after 1–3 months when the tumor reached a volume of 1,500 mm^3^ and further processed as previously described^27^. To explant the tumors, the mice were treated as described above. Tumor tissue and blood were collected, and the animals were sacrificed by exsanguination.

### Approval statement

The study involving primary patient samples was approved by the Ethics Committee of the Canton of Bern, Switzerland (2020–00,498). Ethical approval for all animal experiments was also obtained from the Ethics Committee of the Canton of Bern, and the PDX license (BE85/18) was granted by the Veterinary Service (Veterinärdienst) of the Canton of Bern. The study was conducted in strict accordance with ARRIVE 2.0 guidelines to ensure the ethical and accurate reporting of animal research. All methods were performed in accordance with the relevant guidelines and regulations.

### Immunohistochemistry (IHC)

For immunohistochemical (IHC) analysis LS174T, CaCo2, SW620, and SW480 cells were detached using trypsin and washed once with PBS. Cell pellets were formalin-fixed, paraffin embedded (FFPE), cut into 2 µm sections and mounted on glass slides. Before incubation with CDX2 (EPR2764Y, Cell Marque, Sigma-Aldrich) or E-Cadherin (13–17,000, Thermo Fischer Scientific) antibody, the sections were pretreated (HIER = heat induced epitope retrieval) with pH high/ low buffer to unmask the target antigens, counterstain with Haematoxylin. Slides were stained with BOND RX (Leica Biosystems) and scanned with a Pannoramic 250 Flash II (3DHistech Ltd., Budapest, Hungary) scanner.

### Sequential immunofluorescence and image acquisition

2.5 μm thick FFPE sections underwent simultaneous dewaxing and antigen retrieval protocol on PT Module™ (Epredia). Tissue sections were placed on the rack of PT module and incubated in the Dewax and HIER Buffer H (TA999-DHBH, Epredia) for 1 h at 102 °C. Subsequently, slides were rinsed and stored in Multistaining Buffer (BU06, Lunaphore Technologies) until use. After this preparation step, the sample, reagents, and buffers were loaded on COMET™ (Lunaphore Technologies).

COMET™ performs sequential immunofluorescence (seqIF™), where tissues undergo subsequent cycles of staining, imaging, and antibody elution in a fully automated manner. Staining is performed with standard unconjugated primary antibodies and secondary antibodies labeled with fluorophores. DAPI (Thermo Scientific) is used as counterstaining reagent. Antibodies are listed in Table 1. Image acquisition step is integrated on the device and after imaging, removal of antibodies is achieved with a gentle elution protocol that prepares tissue for the next staining-imaging cycle. At the end of the assay, a single ome.tif file that contains an already stitched, aligned, and stacked image is ready to visualize all the markers and counterstaining. Background subtraction can be applied by Lunaphore Viewer for the detection of distinct signals.

**Table 1:** List of antibodies.

	Antibody	Supplier	Catalog no	Clone
Primary antibodies	CDX2	Cell Marque	235R-16	EPR2764Y
	Cytokeratin	Dako	M3515	AE1/AE3
	E-cadherin	BD Bioscience	610,181	36/E
Secondary antibodies	Alexa Fluor™ Plus 647, goat anti-Rabbit	Lunaphore Technologies	DR647RB	-
	Alexa Fluor™ Plus 647, goat anti-Mouse	Lunaphore Technologies	DR647MS	-
	Alexa Fluor™ Plus 555, goat anti-Mouse	Lunaphore Technologies	DR555RB	-

	**Name**	**Supplier**	**Catalog no**	
Counterstain	DAPI	Thermo Scientific	62,248	

### Digital image analysis

The CRC patient multiplex scan was divided into 100 smaller images of equal size (W: 836.74 μm, H:837.89 μm). We randomly chose 30 images to analyze tumor buds. Images were analyzed by using open-source software QuPath version 0.3.0 (https://qupath.github.io/). DAPI, CDX2, Cytokeratin (CK), and E-cadherin (E-CAD) channels were exported. In QuPath, tumor buds were annotated manually as well as the corresponding nearest primary tumor region. CK was used to facilitate the identification of tumor cells (including tumor buds), i.e. only CK + DAPI + objects were considered. Within these annotated areas, the Stardist algorithm was used for nuclear segmentation based on DAPI (nuclear) staining within QuPath. Additionally, expression measurements for protein markers CDX2, and E-cadherin were acquired. Although CDX2, as a nuclear marker, could be accurately quantified without modification of the Stardist, to correctly estimate E-cadherin (membrane marker) expression adjustments using *cellExpansion* and *cellConstrainScale* were performed. This was done to align the cell segmentation line along the border of the cell for a more accurate measurement of E-cadherin expression level*. *The same approach was used for the PDX model multiplex scan.

### Statistics and data analysis

Statistical analyses were conducted using the PRISM software (GraphPad Software version 10.1.2, San Diego, www.graphpad.com). Nonparametric Mann–Whitney U, for transwell migration and transwell invasion, colony formation and cell count with n > 3, unpaired T-test, for transwell migration and transwell invasion with n = 3, and two-way Anova, for wound healing assay, were applied. For multiplex data analysis, Wilcoxon signed-rank test was applied to determine differences in expression of CDX2, E-cadherin in tumor buds versus mean CDX2, E-cadherin expression in corresponding, nearest tumor regions, respectively. *P* values *p* < 0.05 were considered to be statistically significant.

## Supplementary Information

Supplementary Information 1.

Supplementary Information 2.

## Data Availability

The raw data of this study are included in the supplementary material. Further inquiries can be directed to the corresponding authors.
