# Supplementary material for: CDX2 loss in colorectal cancer cells is associated with invasive properties and tumor budding
Source: Sci Rep. 2025 Jul 6;15:24113. doi: 10.1038/s41598-025-07278-x (PMC12230109; doi:10.1038/s41598-025-07278-x)
Supplement: Supplementary file 1 — Supplementary Information 1. [file 41598_2025_7278_MOESM1_ESM.pptx]

## Slide 1
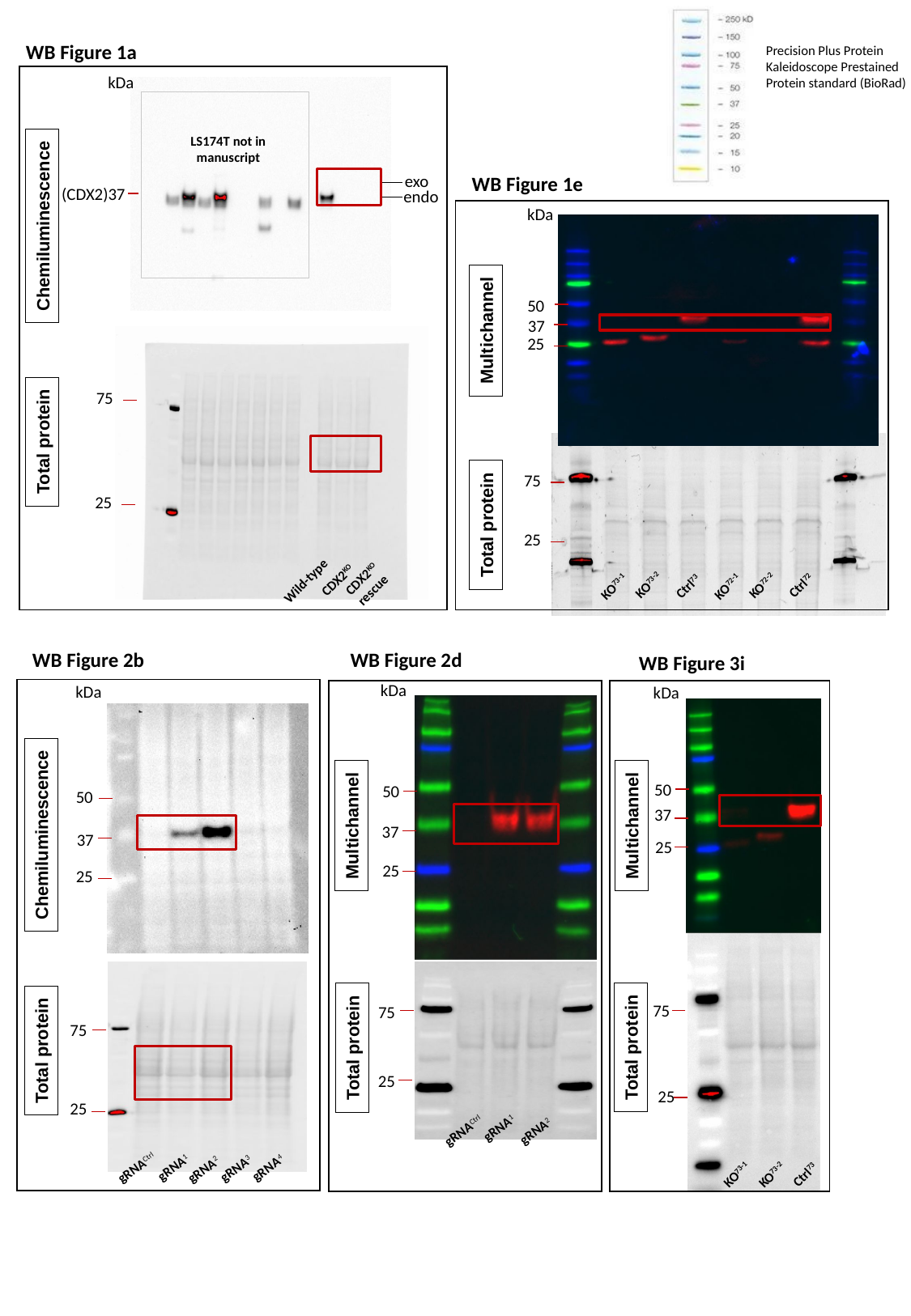

WB Figure 1a
Precision Plus Protein
Kaleidoscope Prestained
Protein standard (BioRad)
kDa
LS174T not in manuscript
exo
WB Figure 1e
(CDX2)37
endo
kDa
Chemiluminescence
50
37
Multichannel
25
75
Total protein
75
25
Total protein
25
CDX2KO rescue
CDX2KO
Wild-type
KO73-2
KO72-2
Ctrl72
Ctrl73
KO72-1
KO73-1
WB Figure 2b
WB Figure 2d
WB Figure 3i
kDa
kDa
kDa
50
50
50
37
Multichannel
Multichannel
37
Chemiluminescence
37
25
25
25
75
75
75
Total protein
Total protein
Total protein
25
25
25
gRNA1
gRNACtrl
gRNA2
gRNA1
gRNACtrl
gRNA4
gRNA3
gRNA2
KO73-1
Ctrl73
KO73-2

## Slide 2
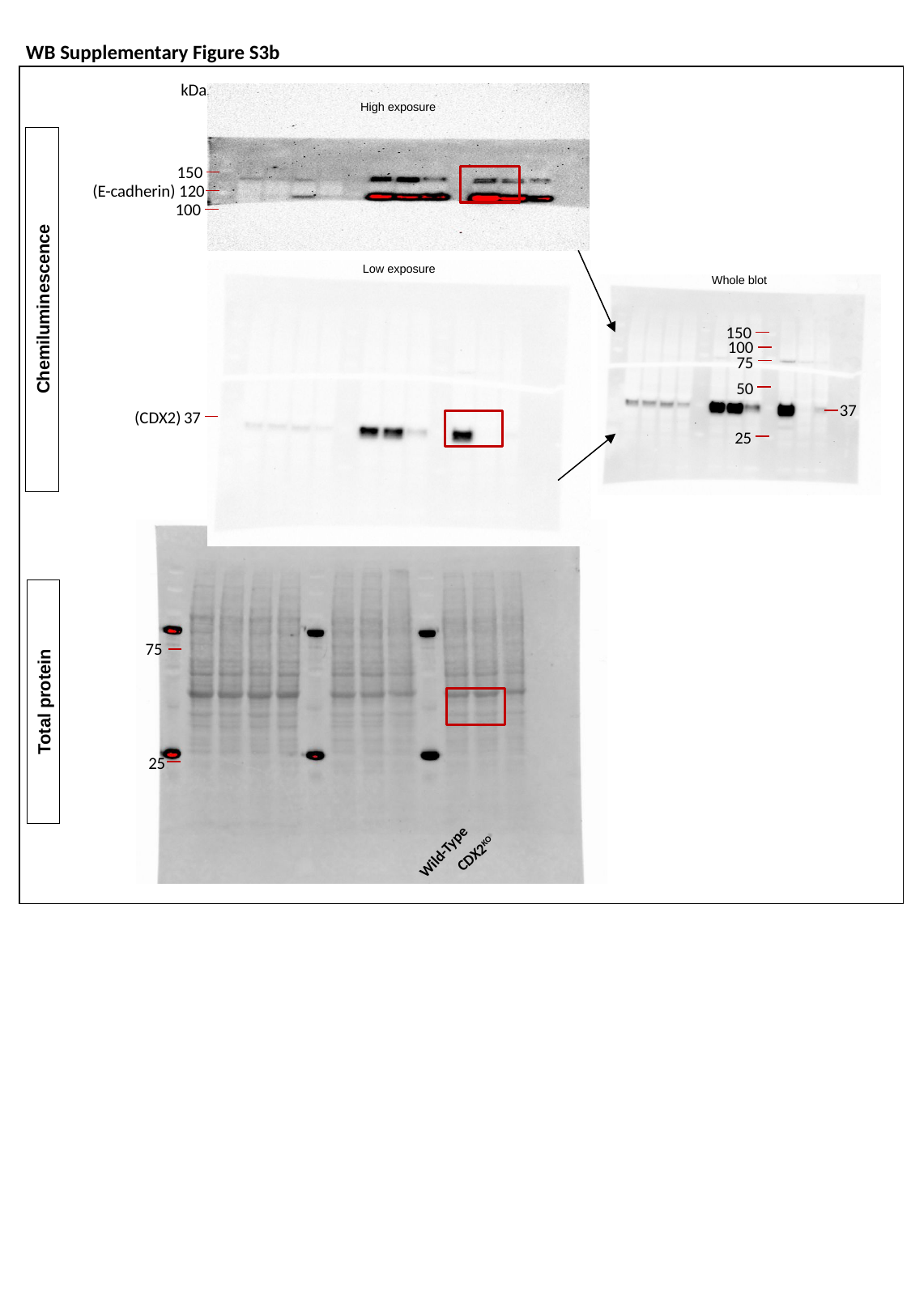

WB Supplementary Figure S3b
kDa
High exposure
150
(E-cadherin) 120
100
Low exposure
Whole blot
Chemiluminescence
150
100
75
50
37
(CDX2)
37
25
75
Total protein
25
Wild-Type
CDX2KO
