# Supplementary material for: CDX2 loss in colorectal cancer cells is associated with invasive properties and tumor budding
Source: Sci Rep. 2025 Jul 6;15:24113. doi: 10.1038/s41598-025-07278-x (PMC12230109; doi:10.1038/s41598-025-07278-x)
Supplement: Supplementary file 2 — Supplementary Information 2. [file 41598_2025_7278_MOESM2_ESM.pptx]

## Slide 1
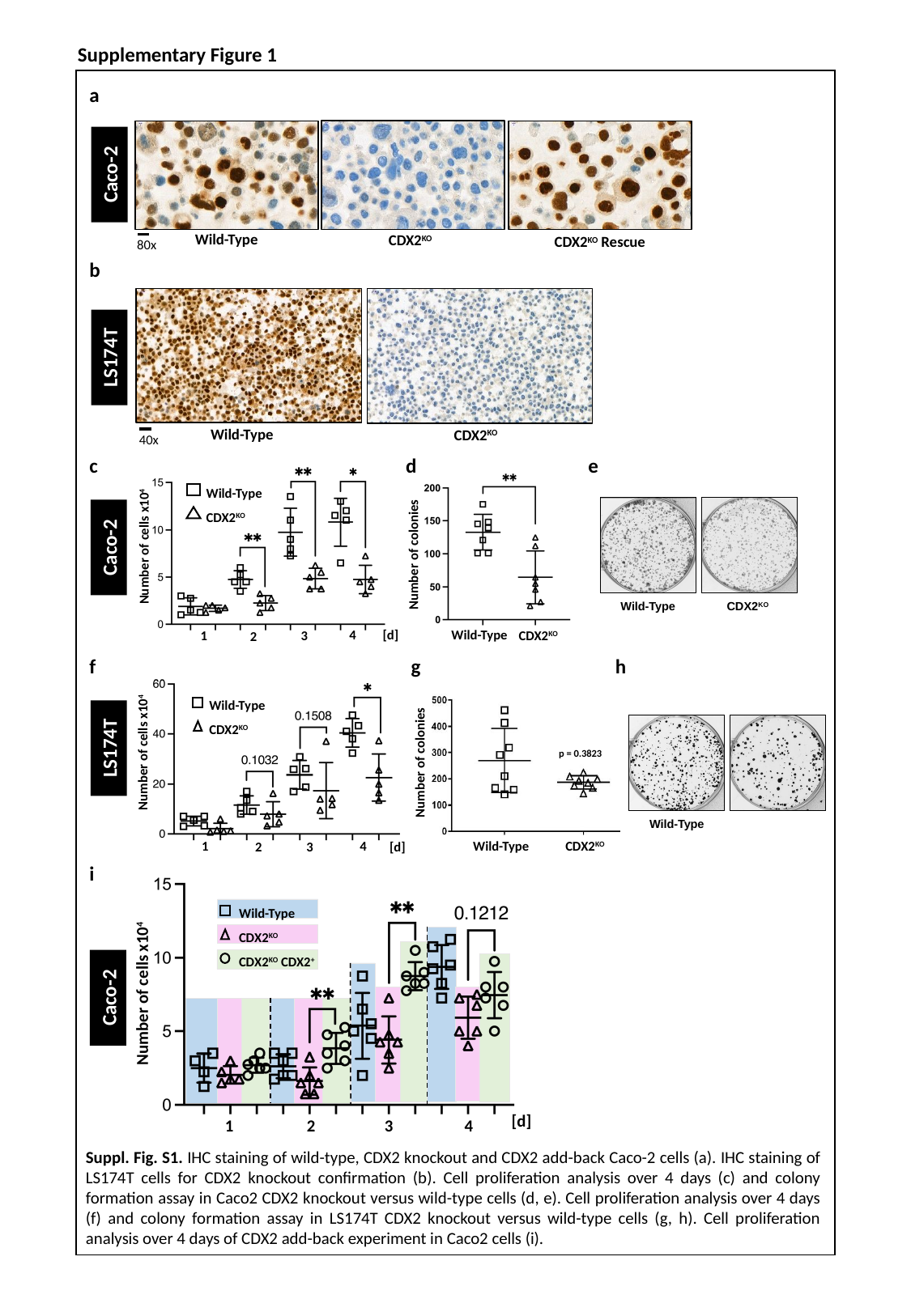

Supplementary Figure 1
a
CDX2KO
Caco-2
d
Wild-Type
CDX2KO
CDX2KO Rescue
80x
b
LS174T
Wild-Type
CDX2KO
40x
e
d
c
Wild-Type
CDX2KO
Caco-2
Number of cells x104
Number of colonies
Wild-Type
CDX2KO
4
Wild-Type
[d]
CDX2KO
3
1
2
f
g
h
Wild-Type
CDX2KO
LS174T
Number of cells x104
p = 0.3823
Number of colonies
Wild-Type
Wild-Type
CDX2KO
1
4
3
2
[d]
i
Wild-Type
CDX2KO
CDX2KO CDX2+
Number of cells x104
Caco-2
[d]
3
2
4
1
Suppl. Fig. S1. IHC staining of wild-type, CDX2 knockout and CDX2 add-back Caco-2 cells (a). IHC staining of LS174T cells for CDX2 knockout confirmation (b). Cell proliferation analysis over 4 days (c) and colony formation assay in Caco2 CDX2 knockout versus wild-type cells (d, e). Cell proliferation analysis over 4 days (f) and colony formation assay in LS174T CDX2 knockout versus wild-type cells (g, h). Cell proliferation analysis over 4 days of CDX2 add-back experiment in Caco2 cells (i).

## Slide 2
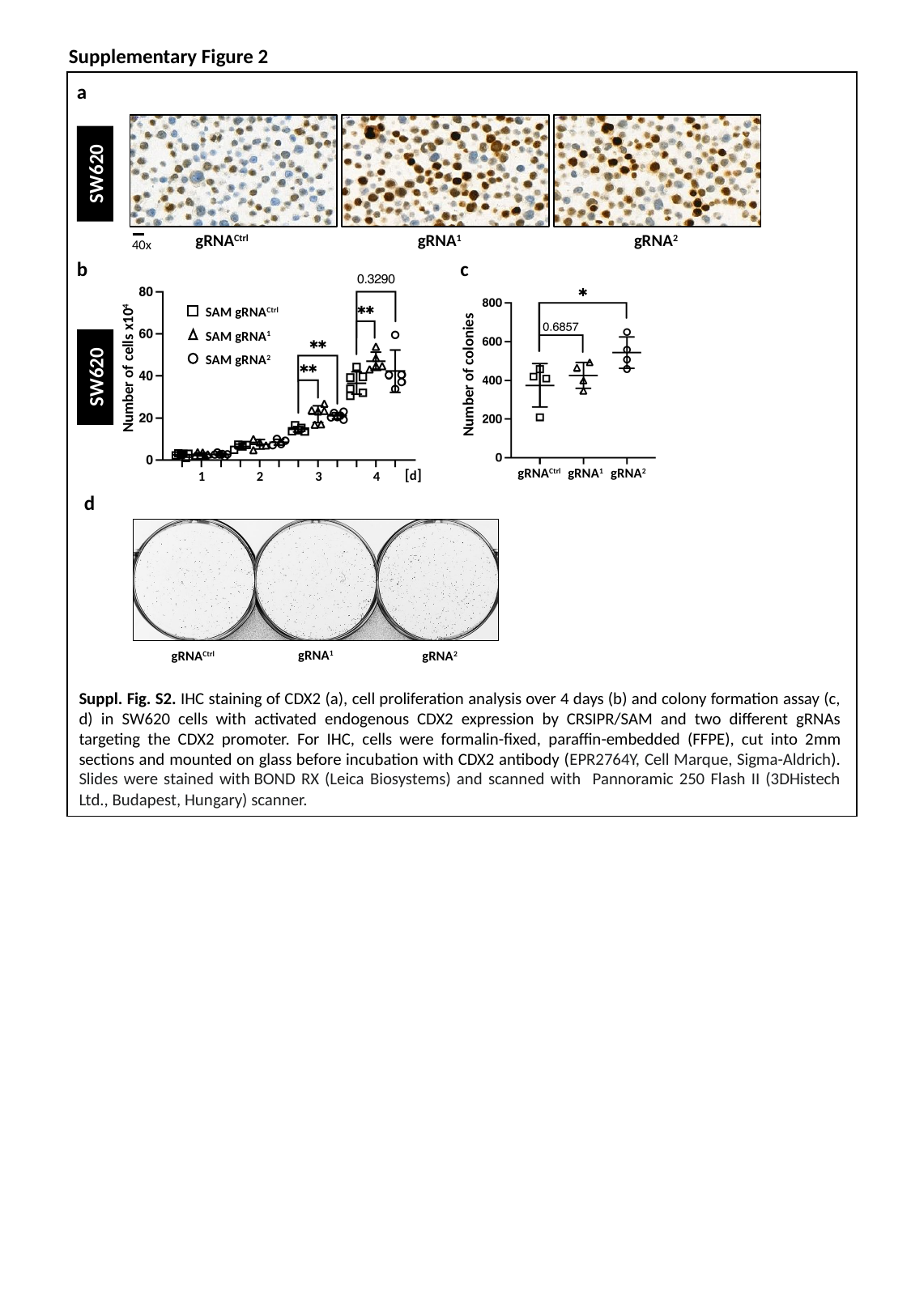

Supplementary Figure 2
a
SW620
gRNA2
gRNA1
gRNACtrl
40x
b
c
SAM gRNACtrl
SAM gRNA1
SAM gRNA2
Number of cells x104
SW620
Number of colonies
gRNACtrl
gRNA1
gRNA2
[d]
1
2
3
4
d
gRNA1
gRNACtrl
gRNA2
Suppl. Fig. S2. IHC staining of CDX2 (a), cell proliferation analysis over 4 days (b) and colony formation assay (c, d) in SW620 cells with activated endogenous CDX2 expression by CRSIPR/SAM and two different gRNAs targeting the CDX2 promoter. For IHC, cells were formalin-fixed, paraffin-embedded (FFPE), cut into 2mm sections and mounted on glass before incubation with CDX2 antibody (EPR2764Y, Cell Marque, Sigma-Aldrich). Slides were stained with BOND RX (Leica Biosystems) and scanned with Pannoramic 250 Flash II (3DHistech Ltd., Budapest, Hungary) scanner.

## Slide 3
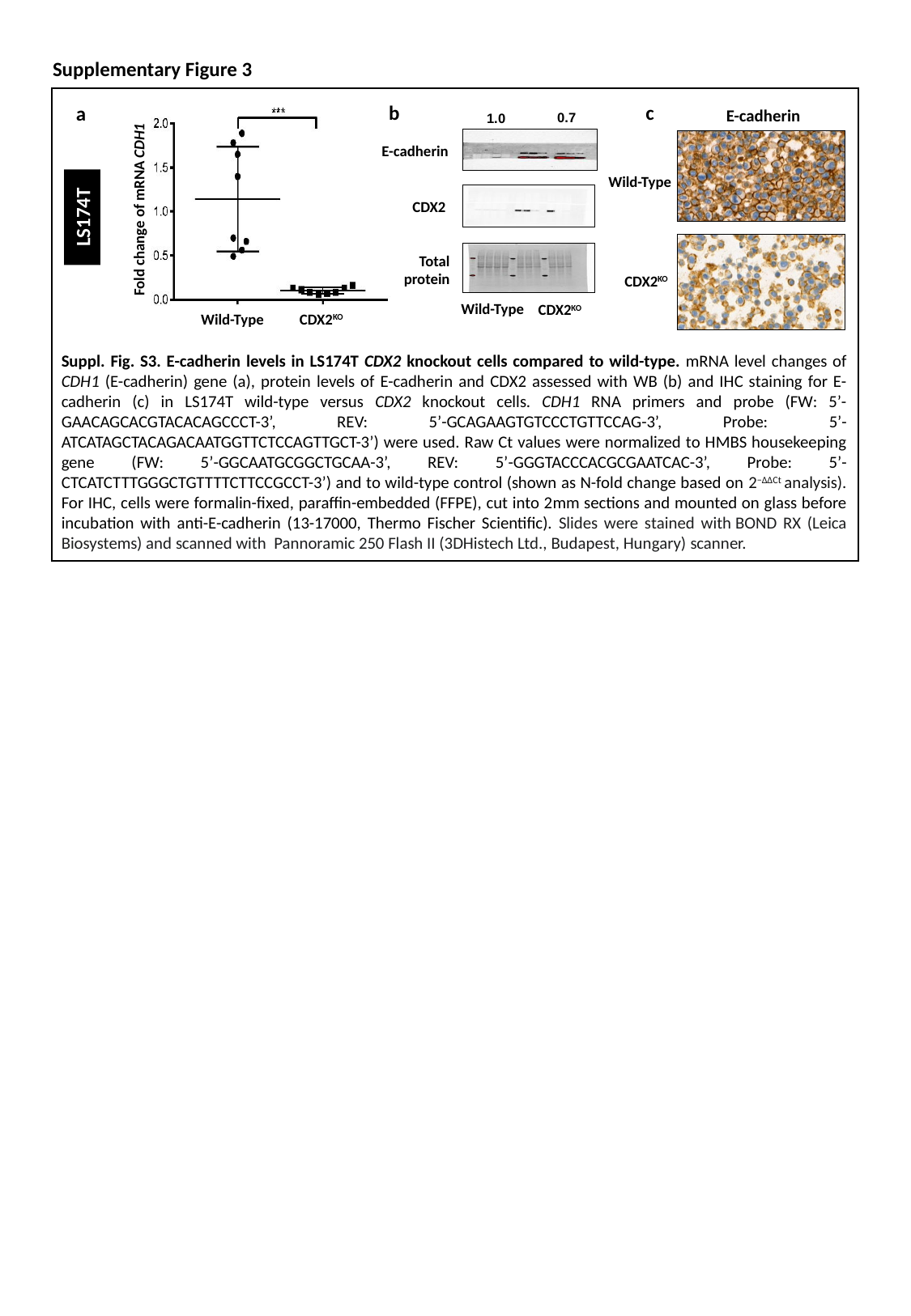

Supplementary Figure 3
b
c
a
E-cadherin
0.7
1.0
E-cadherin
Wild-Type
CDX2
Fold change of mRNA CDH1
LS174T
Total
 protein
CDX2KO
Wild-Type
CDX2KO
Wild-Type
CDX2KO
Suppl. Fig. S3. E-cadherin levels in LS174T CDX2 knockout cells compared to wild-type. mRNA level changes of CDH1 (E-cadherin) gene (a), protein levels of E-cadherin and CDX2 assessed with WB (b) and IHC staining for E-cadherin (c) in LS174T wild-type versus CDX2 knockout cells. CDH1 RNA primers and probe (FW: 5’-GAACAGCACGTACACAGCCCT-3’, REV: 5’-GCAGAAGTGTCCCTGTTCCAG-3’, Probe: 5’-ATCATAGCTACAGACAATGGTTCTCCAGTTGCT-3’) were used. Raw Ct values were normalized to HMBS housekeeping gene (FW: 5’-GGCAATGCGGCTGCAA-3’, REV: 5’-GGGTACCCACGCGAATCAC-3’, Probe: 5’-CTCATCTTTGGGCTGTTTTCTTCCGCCT-3’) and to wild-type control (shown as N-fold change based on 2−ΔΔCt analysis). For IHC, cells were formalin-fixed, paraffin-embedded (FFPE), cut into 2mm sections and mounted on glass before incubation with anti-E-cadherin (13-17000, Thermo Fischer Scientific). Slides were stained with BOND RX (Leica Biosystems) and scanned with Pannoramic 250 Flash II (3DHistech Ltd., Budapest, Hungary) scanner.
